# Lobomycosis in Man and Lobomycosis-like Disease in Bottlenose Dolphin, Venezuela

**DOI:** 10.3201/eid1508.090347

**Published:** 2009-08

**Authors:** Luis Bermudez, Marie-Françoise Van Bressem, Oscar Reyes-Jaimes, Alejandro J. Sayegh, Alberto E. Paniz-Mondolfi

**Affiliations:** Cetacean Research Center, Margarita, Venezuela (L. Bermudez, A.J. Sayegh); Peruvian Centre for Cetacean Research, Pucusana, Peru (M.-F. Van Bressem); Instituto de Biomedicina, Caracas, Venezuela (O. Reyes-Jaimes, A.E. Paniz-Mondolfi); St. Luke’s–Roosevelt–Beth Israel Medical Center, New York, New York, USA (A.E. Paniz-Mondolfi); Instituto Venezolano de los Seguros Sociales, Caracas, Venezuela (A.E. Paniz-Mondolfi)

**Keywords:** Lobomycosis, lacaziosis, lobomycosis-like disease, Tursiops truncatus, skin disease, bottlenose dolphin, Venezuela, fungi, parasites, dispatch

## Abstract

We report 1 case of lobomycosis caused by *Lacazia loboi* in a fisherman and 1 case of lobomycosis-like disease in a bottlenose dolphin (*Tursiops truncatus*) along the coast of Venezuela. These findings suggest that the marine environment is a likely habitat for *L. loboi* and a reservoir for infection.

Lobomycosis (lacaziosis) is a chronic, granulomatous, fungal infection of the skin and subcutaneous tissues that affects humans and members of the family Delphinidae (*1*–*6*). It is caused by the noncultivable yeast-like organism (*Lacazia loboi*) of the order Onygenales (*7*).

Rare in humans, lobomycosis was first reported in Recife, Brazil, in 1930 (*1*) and subsequently in other countries in South and Central America, where it seems to be endemic (*4*). It was also recently reported in Europe, Canada, the United States, and South Africa, mostly in persons who had traveled to Central or South America or had contact with an infected dolphin (*8*–*10*). Geographic and climatic conditions associated with endemic human lobomycosis are those of tropical continental areas that are generally located 200–250 m above sea level and characterized by dense vegetation, annual rainfall <2,000 mm, mean temperature of 24°C, and mean relative humidity >75% (*4*).

Cases of lobomycosis have been found in bottlenose dolphins (*Tursiops truncatus*) and Guiana dolphins (*Sotalia guianensis*) in North and South America since the 1970s and are being increasingly reported (*2*,*3*,*5*,*6*). The disease in these mammals is characterized by white to pink, verrucous lesions, often in pronounced relief that may ulcerate and form large plaques. Lobomycosis-like disease, a syndrome pathoanatomically consistent with lobomycosis but for which a histologic diagnosis is lacking, has been found in coastal bottlenose dolphins from Colombia, Ecuador, Peru, and Brazil, Guiana dolphins in Brazil (*5*,*11*), and Indo-Pacific bottlenose dolphins (*T*. *aduncus*) in the tropical lagoon of Mayotte in the Indian Ocean (*12*).

*L*. *loboi* cells from lesions in bottlenose dolphins were smaller than those found in infected tissues in humans, which suggests that the organism may not be identical in the 2 hosts (*13*). Serologic data have indicated that dolphins and humans are infected with similar *L*. *loboi* strains (*14*). The possibility of humans acquiring lobomycosis from dolphins appears low; only 1 documented case of disease transmission from a rescued dolphin to its attendant occurred in the early 1970s (*9*). The ecology of lobomycosis in humans and odontocetes seems to be unconnected. Infections occur mostly in persons inhabiting the Amazon Basin and in inshore and estuarine dolphins in North and South America (*3*–*6*). We report cases of lobomycosis in a fisherman and lobomycosis-like disease in a bottlenose dolphin along the coast of Venezuela.

## The Cases

### Human

**Figure 1:**
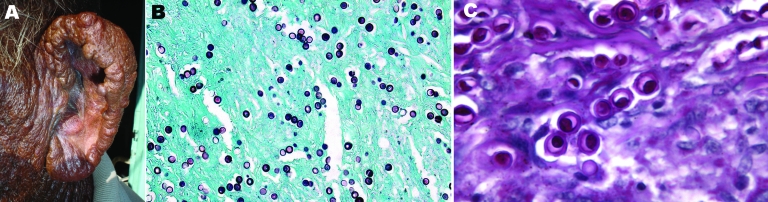
A) Multiple, confluent, keloid-like, hyperchromic nodules with flat shiny surfaces involving the entire free border, posterior aspect, and lobule of the left ear of a fisherman, Venezuela. B) Numerous *Lacazia loboi* tissue-phase organisms within the stroma. Note the typical chain pattern showing simple gemation budding (Gomori-Grocott stain, magnification ×100). C) Yeast cells showing typical double refraction of the membrane and protoplasmic bodies within cells (periodic acid–Schiff stain, magnification ×600).

A 62-year-old fisherman from the central coast of Venezuela (Puerto Cruz and Chichiriviche de la Costa, 10°32′N, 67°14′W) was examined during a fieldwork expedition in March 2008. He had extensive lesions on the left ear and recalled that his illness began when he was ≈52 years of age and had accidentally injured the posterior portion of the helix of that ear with a fishhook. A small, solitary, hard nodule subsequently developed and was later accompanied by similar satellite lesions that tended to become confluent and form harder nodules, sometimes hyperchromic with flat and shiny surfaces (Figure 1, panel A). These nodules slowly invaded the entire free border, posterior aspect, and lobule of the ear and caused occasional pruritus and a tingling sensation. Because of the diffuse infiltration of the ear, the condition was initially diagnosed as diffuse cutaneous leishmaniasis or lepromatous leprosy.

Microscopic findings included a granulomatous reaction indicated by lymphohistiocytic elements with large numbers of multinucleated Langhan-type giant cells and numerous isolated yeast-like organisms with a birefringent membrane isolated or in chains alternating with some piriform elements showing simple gemation (Figure 1, panels B, C). The patient refused otoplasty and was treated with itraconazole; some nodules partially regressed.

### Dolphin

**Figure 2:**
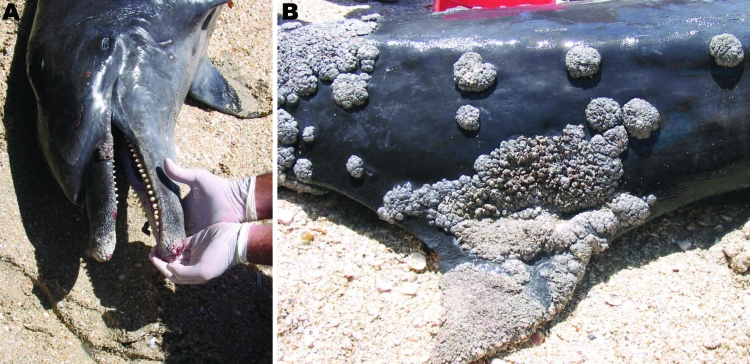
Extensive lobomycosis-like disease on the beak (A) and dorsal fin (B) of a bottlenose dolphin (*Tursiops truncatus*) stranded on Margarita Island, Venezuela.

On June 28, 2004, an adult male, likely inshore, bottlenose dolphin, which had recently died, was found on a beach of La Restinga National Park (11°01′N, 64°10′W) on Margarita Island, Venezuela. The dolphin was 3.8 m long and was emaciated. Several teeth were missing, especially at the distal end of the beak, and an 8-cm *Conchoderma auritum* stalked barnacle was attached to the right 10th mandibular tooth. The dolphin had severe lobomycosis-like disease with a large number of white, gray, and pink proliferating, congregating lesions, some bleeding, with keloidal and verrucous characteristics that formed rosettes on the beak, back, flanks, dorsal fin, tailstock, and tail (Figure 2). The dorsal fin was severely affected and the asymmetric distribution of the lesions caused the fin to bend. Granulomas extended into the oral cavity between the maxillar teeth and the palate. Unfortunately, because of a variety of factors, including a lack of field sampling capabilities, presence of crowd, and limited beach access for transport, no necropsy was conducted and no samples were available. However, the severe emaciation suggested that the dolphin had a chronic debilitating disease. Whether its poor health status favored the wide dissemination of lobomycosis-like disease or whether lobomycosis-like disease was the primary undermining factor remains unknown.

## Conclusions

We report lobomycosis in a fisherman and lobomycosis-like disease in a bottlenose dolphin along the coast of Venezuela. The fisherman likely contracted the disease from the marine environment after pathogen inoculation with a fishing hook. He had never visited the Amazon Basin. Although the human and dolphin cases were probably not related, they suggest the role of the marine environment as a likely natural habitat for *L*. *loboi* and as a reservoir for infection. Along the central coasts of Venezuela and Margarita Island, temperatures range from 22°C to 28°C, annual rainfall ranges from 0 mm to 500 mm (Margarita Island) or >500 mm (central coast), and the mean relative humidity is ≈50%.

Many aspects of transmission, pathogenesis, and ecology of lobomycosis are still poorly understood. Transmission of lobomycosis among Delphinidae may occur by contact, as suggested by the endemic status of the disease in bottlenose dolphins in the Indian River Lagoon in Florida, USA, and possible transmission from mother to calf in an Indo-Pacific bottlenose dolphin from the Mayotte Lagoon (*5*,*12*). Humans may also acquire the infection through rare contact with infected free-ranging Delphinidae. The disease signs and pathologic changes are similar in humans and dolphins. In humans, lobomycosis is associated with an apparent partial deficit of cell-mediated immunity and no alterations of humoral immunity (*15*). In dolphins, the disease is related to a substantial decrease in CD4+ helper T-lymphocytes and CD19+ and CD21+ B cells (*6*). Lesions are also similar in humans and cetaceans, although they tend to be larger in cetaceans. These lesions cover a wide and pleiomorphic clinical spectrum, ranging from the typical smooth and shiny nodular lesions with keloidal aspect to the extensive and confluent verrucous lesions. They occur predominately on the most exposed and cooler areas (*4*,*6*): i.e., head, back, dorsal fin, flanks, caudal peduncle, and tail in dolphins; and lower limbs, outer ears, upper limbs, and face in humans.

The apparent emergence of lobomycosis, lobomycosis-like disease, and other skin diseases in coastal cetaceans from South America and the Indian Ocean (*5*,*11**,**12*) is cause for concern. This emergence may be indicative of increased biological contamination and environmental changes, including climatic changes worldwide, which may represent a potential threat to human health.
